# The association between COVID‐19 and incident gestational diabetes (GDM): A population‐based case–control study of the National Health Insurance Research Database in Taiwan

**DOI:** 10.1111/jdi.70228

**Published:** 2025-12-28

**Authors:** Liang‐Yu Lin, Chi‐Jen Chen, Mei‐Huei Chen, Chun‐Wei Chen, Pau‐Chung Chen

**Affiliations:** ^1^ Institute of Environmental and Occupational Health Science National Taiwan University Taipei Taiwan; ^2^ National Taiwan University Hospital Taipei Taiwan; ^3^ Institute of Epidemiology and Preventive Medicine National Taiwan University Taipei Taiwan; ^4^ Institute of Population Health Science, National Health Research Institutes Zhunan Taiwan; ^5^ National Taiwan University Hospital Hsin‐Chu Branch Hsinchu Taiwan; ^6^ National Institute of Environmental Health Sciences, National Health Research Institutes Zhunan Taiwan

**Keywords:** COVID‐19, electronic health records, gestational diabetes, long COVID

## Abstract

**Background:**

Reports suggested that diabetes could be a complication arising from COVID‐19; however, the relationship between COVID‐19 and the development of gestational diabetes mellitus (GDM) remains unclear.

**Objectives:**

This study aimed to investigate the association between COVID‐19 infections and the risk of incident GDM in pregnant women.

**Methods:**

We analyzed data from Taiwan's National Health Insurance Research Database (NHIRD), which is linked to the Birth Reporting Database and the COVID‐19 testing database between 2020 and 2022. A case–control study was conducted, matching pregnant women by age and region. We employed multivariable logistic regression, adjusting for matching factors and potential confounders. The findings were further validated through a sensitivity analysis using a cohort design with landmark analysis.

**Results:**

The study included 134,375 pregnant women, comprising 26,875 GDM cases and 107,500 matched controls. After adjusting for covariates, we found no evidence supporting an association between prior COVID‐19 infection and incident GDM (adjusted odds ratio [aOR] = 0.95, 95% confidence interval [CI] = 0.89–1.01). Notably, some evidence showed that receiving at least one COVID‐19 vaccination was associated with a decreased risk of GDM (aOR = 0.90, 95% CI = 0.87–0.93). These results remained consistent in the sensitivity analysis.

**Conclusion:**

Despite COVID‐19 now being endemic with less virulent variants, ongoing vigilance regarding potential pregnancy‐related impacts of SARS‐CoV‐2 is essential. It is also critical to promote vaccination among women of childbearing age, and further research is necessary to explore COVID‐19‐related complications during pregnancy.

## Graphical abstract

Our study found no evidence that COVID‐19 infection is linked to gestational diabetes; however, it shows some evidence of an association between vaccination and a reduced risk.

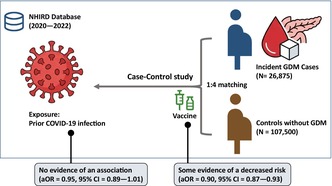

## INTRODUCTION

Despite being endemic in many countries, the long‐term sequelae of COVID‐19 remain a significant public health concern. Most people recover from COVID‐19 within 4 weeks, but some people continue to experience persistent or newly developed conditions for over 3 months, known as “post‐COVID syndrome” or “long COVID.”^1^. Common long COVID symptoms include fatigue, dyspnea, and cognitive impairment; however, some people with long COVID may have other complications, such as diabetes. A study analyzing an electronic health records (EHRs) database in Hong Kong reported that the risk of developing incident type 2 diabetes increased after COVID‐19 infections (hazard ratio (HR) = 1.23, 95% confidence intervals (CI) = 1.15–1.30)^2^. Another study analyzing EHRs in England also reported that the hazards of incident type 2 diabetes increased within 2 years after COVID‐19 infection (week 1–4: HR = 4.30, 95% CI = 4.06–4.55; week 53–102: HR = 1.24, 95% CI = 1.14–1.35)^3^.

In addition to type 1 and type 2 diabetes, another form of diabetes is gestational diabetes (GDM), defined as glucose intolerance that first develops during pregnancy^4^. Globally, the prevalence of GDM in 2021 was estimated to be around 14% (95% CI: 13.96%–14.04%), which was similar across different regions^5^. Although the hyperglycemia is transient, approximately half of women with GDM may develop persistent diabetes later in life^6^. In addition, people with GDM would experience an increased risk of adverse maternal and birth outcomes, such as preterm delivery, pre‐eclampsia, and large or small for gestational age^7^. Regarding the possible risk factors of GDM, some studies explored the potential association between COVID‐19 and GDM, but the results are inconclusive. A recent study using claim data in the US reported some evidence that COVID‐19 infections during pregnancy were associated with an increased risk of GDM (risk ratio (RR): 1.12; 95% CI: 1.08–1.15)^8^. However, in the English study previously mentioned, in the subgroup analysis, the authors did not observe an increase in the incidence of GDM after COVID‐19 diagnosis among female participants^3^. In addition, as shown in Figure S1, these cohort studies may be affected by immortal time bias, because none of the study participants will be diagnosed with GDM until gestational week 24^9^, ^10^, ^11^, ^12^.

Because both COVID‐19 and GDM impose severe health consequences on maternal health, understanding their possible association accurately will improve maternal care, which could inform healthcare policy regarding infection control. Consequently, by conducting a population‐based study, we aim to investigate the association between COVID‐19 infections and the risk of incident GDM.

## METHODS

**Figure 1:**
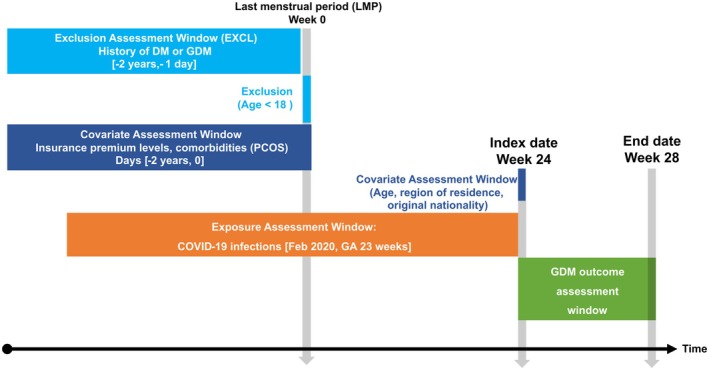
Illustration of study design and variable assessment time windows. GA, gestational weeks; GDM, gestational diabetes.

We conducted a population‐based case–control study using Taiwan's National Health Insurance Research Database (NHIRD) between February 2020 and December 2022. The overview of the study design and variable assessment time windows is summarized in Figure 1.

### Data source

In Taiwan, all residents living for more than six months and newborns have been required to join the National Health Insurance (NHI) since 1995. In 2022, 92.4% of healthcare facilities were contracted with NHI, covering more than 99.8% of the Taiwanese population^13^, ^14^. The reimbursement claim data of NHI, such as outpatient clinic, inpatient hospitalization records, or emergency room visits, were de‐identified and released as NHIRD from 2000. In addition, NHIRD can be linked to other special datasets such as the National Birth Reporting Database, the cancer registry, or the death registry^15^.

### Study population, inclusion, and exclusion criteria

Gestational diabetes can be diagnosed after 24 weeks of gestation^16^, so we only included adult women (≥18 years old) with pregnancies of more than 24 weeks for analysis. Pregnant women were identified from the NHIRD National Birth Reporting Database (Health‐09), which records all mothers and newborn pairs in Taiwan. We linked these pregnant women to their inpatient and outpatient records and estimated their last menstrual period date (LMP) by subtracting gestational week from the date of delivery. We excluded those having existing type 1 or type 2 diabetes mellitus, diabetic ketoacidosis (DKA), or GDM in past pregnancies before LMP. All diagnoses and procedures were identified using a pre‐defined codelist in the International Classification of Diseases, 10th Revision, Clinical Modification (ICD‐10‐CM).

### COVID‐19 exposure ascertainment

Exposure to COVID‐19 was determined by linking the COVID‐19 Vaccination and Diagnoses Database (Health‐102) to NHIRD. This COVID‐19 dataset includes mandatory reports of COVID‐19 rapid antigen tests or PCR results from healthcare services between 2020 and 2022. In addition, we also identified additional COVID‐19 cases from their outpatient or inpatient records using a predefined COVID‐19 diagnostic codelist. Pregnant women with any documented COVID‐19 infection records before the 23rd gestational week were classified as “had COVID‐19 infection,” and the others were labeled as “did not have COVID‐19 infection.”

### Gestational diabetes (GDM) cases and controls ascertainment

We defined pregnant women with incident GDM diagnoses first reported between week 24 and week 28 as cases, inconsistent with the clinical guidelines^16^, ^17^ and the government‐funded prenatal examination Scheme^18^. These people with GDM were identified using an ICD‐10‐CM codelist that included all codes beginning with “O24”. To enhance the diagnostic validity, we only included women with at least one GDM diagnostic code in their hospital admission records or emergency room visits or two consensus GDM diagnoses in their outpatient clinic visit records^19^. Control groups were pregnant women who did not have GDM diagnoses between week 24 and week 28 who were matched to each case in a 4:1 ratio based on age at pregnancy and region of residence^20^.

### Covariates ascertainment

Potential confounders were defined according to previous literature, and covariates to be included in statistical analyses were selected based on a directed acyclic graph (DAG) (Figure S2). We included age, region, ethnicity, socioeconomic status, and a history of polycystic ovarian syndrome (PCOS) in our model. Information on ethnicity was not recorded in NHIRD, so we used mothers' nationality from the Birth Reporting Database. Nationality was further categorized as Taiwanese and non‐Taiwanese. Regarding socioeconomic status, we use the insurance premium quintile as a proxy. A history of PCOS was identified if a person had an ICD‐10‐CM diagnostic code E28.2 within 2 years before the index date (Figure S2).

### Statistical analyses and sensitivity analysis

We first summarized and compared baseline demographic characteristics among GDM cases and their matched controls. Categorical variables were compared using the Chi‐square test, and continuous variables were compared using the *t*‐test. Subsequently, we assessed the association between COVID‐19 infections and incident GDM using logistic regression, adjusting for all covariates.

Sensitivity analyses were conducted to further confirm our results. We first stratified our analysis by vaccination status to compare the differences in effect sizes. Additionally, we conducted a matched cohort study with landmark analysis as a sensitivity analysis to examine our results. In brief, we matched each pregnant woman with a history of COVID‐19 infection to four non‐COVID comparators by age and region. To avoid involving an immortal follow‐up time, we started following them from the first date of gestational week 24 (landmark)^9^. We applied a Cox regression model adjusting for matching factors and covariates to assess the association between COVID‐19 infection and GDM. All data analysis was conducted using SAS 9.4 (SAS Institute Inc., Cary, NC, USA), and data visualization was finalized using R version 4.3.3 (2024‐02‐29 ucrt).

### Ethics approval

This study has been approved by the Institutional Review Board of the National Health Research Institutes (No: EC1120508‐E). We reported our findings according to the RECORD reporting guideline (Table S1)^21^, and attached our study protocol to the Supplementary Material. Our study followed the principles of the Declaration of Helsinki^22^.

## RESULTS

### Distribution of demographic characteristics of the study population

**Figure 2:**
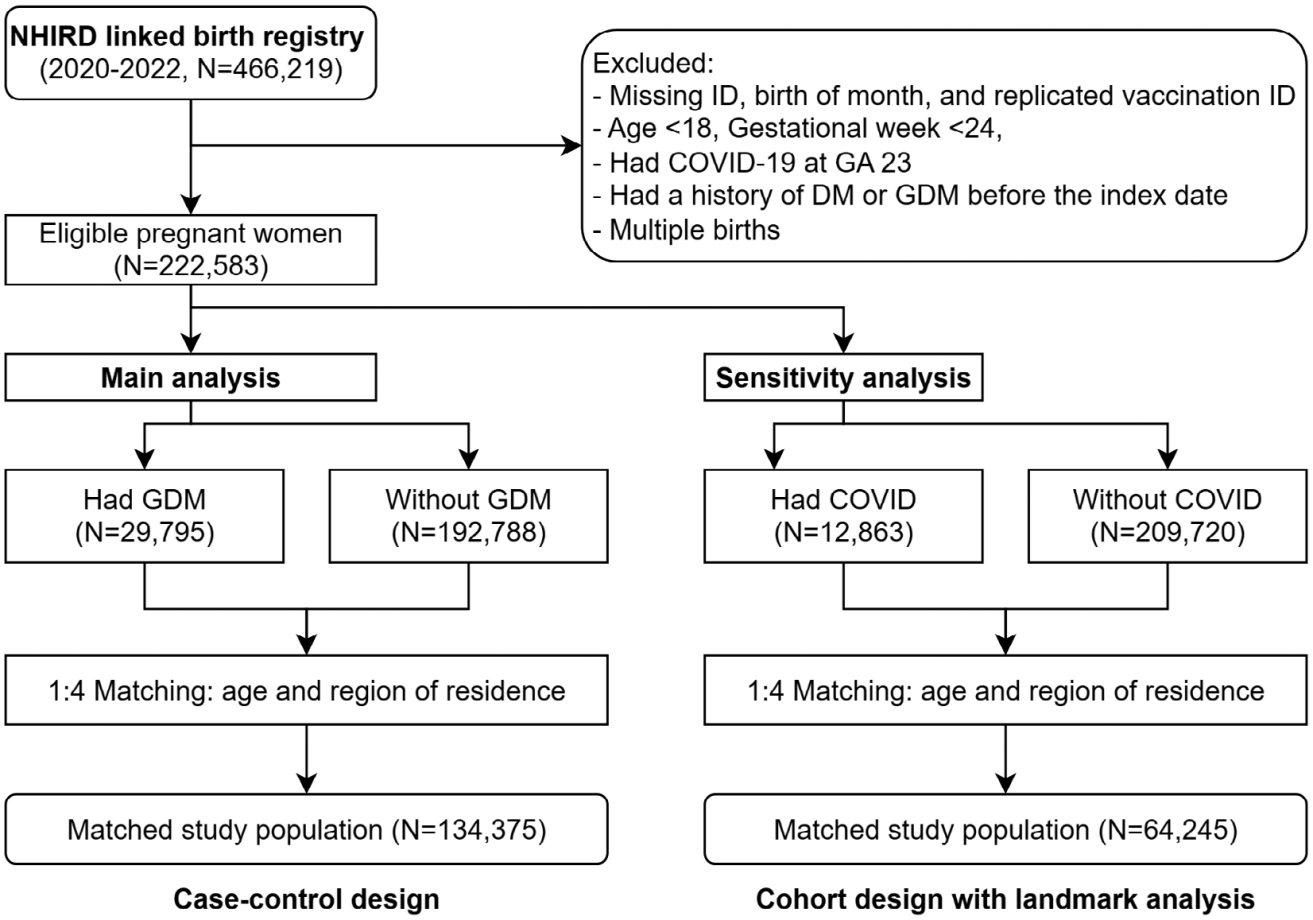
The flow chart of selecting the study population.

The process of selecting the study population was summarized in Figure 2. We identified 134,375 eligible pregnant individuals from NHIRD and the National Birth Reporting database, comprising 26,875 cases with GDM and 107,500 controls without GDM. The distribution of demographic factors was summarized in Table 1. In brief, the mean maternal age at LMP was 32.6 (SD: 4.8) years old. The proportion of COVID‐19 infection was among GDM cases (5.09%) compared to controls (5.64%, *P* < 0.01), and the COVID‐19 vaccination coverage was slightly lower among cases (30.96%) than controls (32.87%, *P* < 0.01). Morbid obesity and PCOS were more prevalent among GDM cases (morbid obesity: 0.62%; PCOS: 4.02%) than controls (morbid obesity: 0.33%; PCOS: 2.65%). Most participants were Taiwanese nationals (92.23%), with a higher proportion among GDM cases (93.31%) compared to controls (91.96%, *P* < 0.01). The distribution of age, regions, and income was balanced across cases and controls (Table 1).

**Table 1:** The distribution of demographic factors of the study population by gestational diabetes (GDM) diagnoses

	Total *N* = 134,375	Had GDM *N* = 26,875	Without GDM *N* = 107,500
Mean age at the last menstrual period	32.6 (4.8)	32.6 (4.8)	32.6 (4.8)
COVID‐19 infections			
Ever had COVID‐19 (total)	7,434 (5.53%)	1,369 (5.09%)	6,065 (5.64%)
History of COVID‐19 before LMP	281 (3.78%)	54 (3.94%)	227 (3.74%)
History of COVID‐19 after LMP	7,153 (96.22%)	1,315 (96.06%)	5,838 (96.26%)
COVID‐19 vaccinations			
Not vaccinated	90,719 (67.51%)	18,554 (69.04%)	72,165 (67.13%)
Vaccinated (≥1 dose)	43,656 (32.49%)	8,321 (30.96%)	35,335 (32.87%)
Morbid obesity			
Not obese	133,857 (99.61%)	26,708 (99.38%)	107,149 (99.67%)
Obese	518 (0.39%)	167 (0.62%)	351 (0.33%)
Polycystic ovarian syndrome (PCOS)			
No	130,441 (97.07%)	25,795 (95.98%)	104,646 (97.35%)
Yes	3,934 (2.93%)	1,080 (4.02%)	2,854 (2.65%)
Region of residence			
Taipei City	14,470 (10.77%)	2,894 (10.77%)	11,576 (10.77%)
New Taipei City	19,715 (14.67%)	3,943 (14.67%)	15,772 (14.67%)
Keelung City	1,345 (1%)	269 (1%)	1,076 (1%)
Taoyuan City	13,380 (9.96%)	2,676 (9.96%)	10,704 (9.96%)
Hsinchu City	3,320 (2.47%)	664 (2.47%)	2,656 (2.47%)
Hsinchu County	4,040 (3.01%)	808 (3.01%)	3,232 (3.01%)
Miaoli County	2,705 (2.01%)	541 (2.01%)	2,164 (2.01%)
Nantou County	2,180 (1.62%)	436 (1.62%)	1,744 (1.62%)
Taichung City	18,125 (13.49%)	3,625 (13.49%)	14,500 (13.49%)
Yunlin County	4,300 (3.2%)	860 (3.2%)	3,440 (3.2%)
Chiayi City	1,330 (0.99%)	266 (0.99%)	1,064 (0.99%)
Chiayi County	2,705 (2.01%)	541 (2.01%)	2,164 (2.01%)
Changhua County	8,435 (6.28%)	1,687 (6.28%)	6,748 (6.28%)
Tainan City	11,800 (8.78%)	2,360 (8.78%)	9,440 (8.78%)
Kaohsiung City	17,990 (13.39%)	3,598 (13.39%)	14,392 (13.39%)
Pingtung County	3,625 (2.7%)	725 (2.7%)	2,900 (2.7%)
Yilan County	2,095 (1.56%)	419 (1.56%)	1,676 (1.56%)
Hualien County	1,460 (1.09%)	292 (1.09%)	1,168 (1.09%)
Taitung County	450 (0.33%)	90 (0.33%)	360 (0.33%)
Penghu County	340 (0.25%)	68 (0.25%)	272 (0.25%)
Kinmen County & Lienchiang County (Matsu)	565 (0.42%)	113 (0.42%)	452 (0.42%)
Income (Taiwan dollars)			
<23,800	38,283 (28.49%)	7,458 (27.75%)	30,825 (28.67%)
23,801–27,600	32,918 (24.5%)	6,679 (24.85%)	26,239 (24.41%)
27,601–40,100	29,840 (22.21%)	5,905 (21.97%)	23,935 (22.27%)
≥40,101	33,334 (24.81%)	6,833 (25.43%)	26,501 (24.65%)
Original nationality			
Taiwan	123,937 (92.23%)	25,078 (93.31%)	98,859 (91.96%)
Non‐Taiwanese	10,438 (7.77%)	1,797 (6.69%)	8,641 (8.04%)

### Comparing the association between COVID‐19 infection history and incident GDM

**Figure 3:**
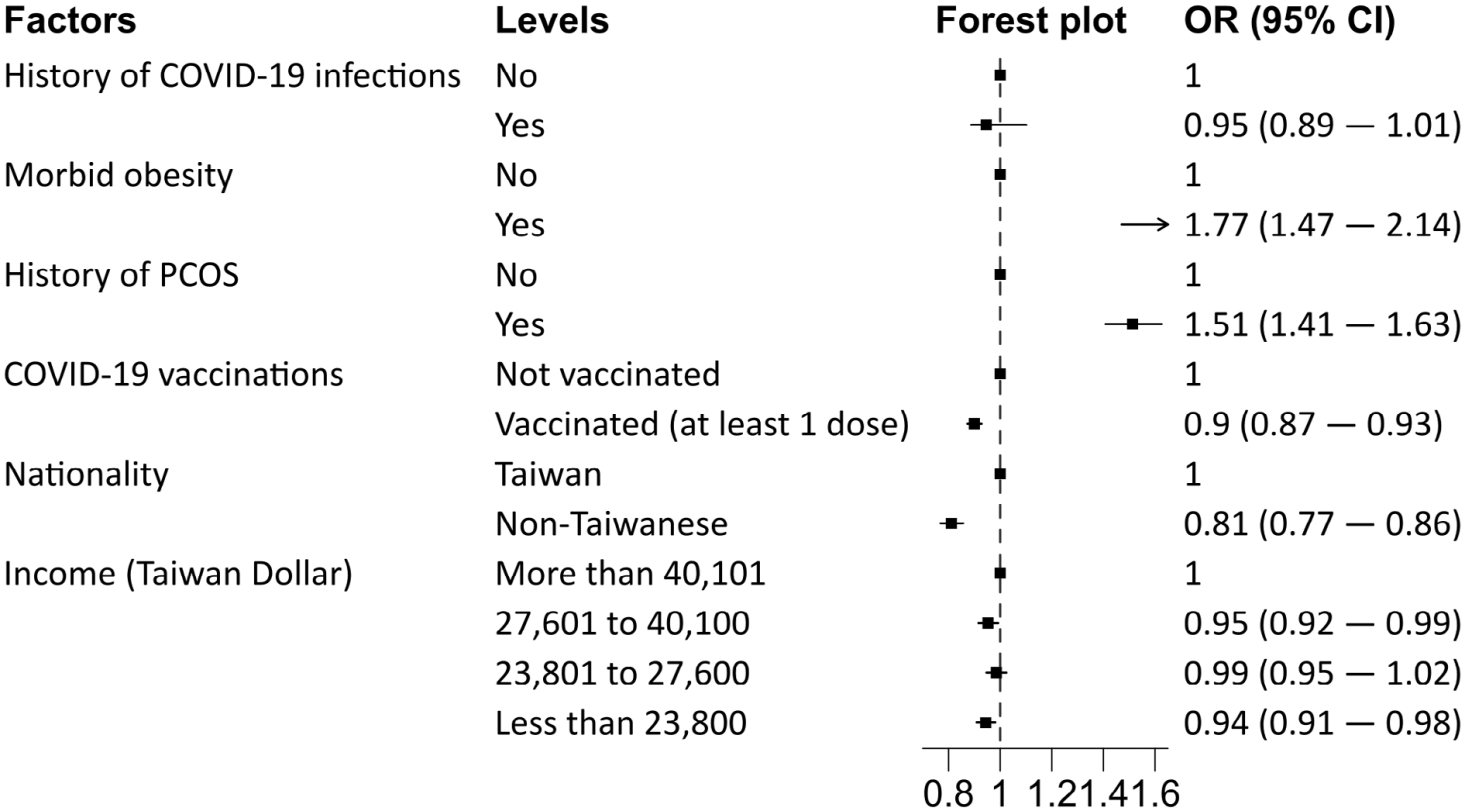
The association between COVID‐19 infections and incident GDM. The model adjusted for age, region of residence, morbid obesity, PCOS, nationality, income, and COVID‐19 vaccination.

The association between COVID‐19 exposure and incident GDM is summarized in Figure 3. After adjusting for matching factors and covariates, we found no evidence that prior COVID‐19 infections were associated with an increased risk of incident GDM (adjusted odds ratio (aOR) = 0.95, 95% CI = 0.89–1.01). As expected, several covariates, such as morbid obesity and PCOS, showed strong associations with an increased risk of GDM. In addition, there was weak evidence that receiving at least one vaccination dose was associated with a reduced risk of GDM (aOR = 0.90, 95% CI = 0.87–0.93). In addition, non‐Taiwanese nationality was also associated with a weak decrease in GDM risk (aOR = 0.81, 95% CI = 0.77–0.83), while there were no consistent associations between income and incident GDM (Figure 3).

### Sensitivity analysis

We used a cohort design with landmark analysis in the sensitivity analysis. The distribution of the demographic characteristics was summarized in Table S2. Among 64,245 pregnant individuals, 12,849 (20.0%) had a history of COVID‐19 infections by gestational week 24. At the same time, the proportion of GDM was slightly lower among the COVID‐19 exposure group (11.52%) compared to the non‐COVID group (13.06%) (Table S2). Similar to the case–control design, after adjusting for matching factors and covariates, we found no evidence that prior COVID‐19 infections were associated with an increased risk of incident GDM using a cohort design with landmark analysis (adjusted HR = 0.95, 95% CI = 0.89–1.02) (Figure S3). In the analysis stratified by vaccination status, we found no evidence that COVID‐19 infection was associated with GDM regardless of vaccination status (Table S3).

## DISCUSSION

In this study, by assessing a national representative birth database, we found no evidence of a significant association between COVID‐19 infection history and GDM among pregnant women between 2020 and 2022 in Taiwan. In addition, there was some evidence that receiving at least one dose of the COVID‐19 vaccine and being of non‐Taiwanese nationality were associated with a decreased risk of GDM incidents. These findings were further confirmed by the sensitivity analysis using a cohort design with landmark analysis.

Our study did not find any evidence between COVID‐19 infection and incident GDM, which differs from previous studies in the US, which showed a positive association between COVID‐19 infection and GDM^8^, but are similar to the results in England^3^. Several factors may contribute to these discrepancies. First, healthcare systems and pandemic responses varied across countries, and the circulating COVID‐19 variants also differed. Unlike the US, Taiwan and the UK both have single‐payer universal healthcare systems, which can facilitate a centralized pandemic response^23^. Taiwan's stringent border controls and universal mask mandates delayed widespread community transmission without requiring a strict lockdown^24^. In mid‐2021, Taiwan experienced a small COVID‐19 outbreak attributed to the Alpha variant. From April 2022 to March 2023, the country experienced three waves of nationwide community transmission, all driven by Omicron sub‐lineages, including BA.2, BA.5, and BA.2.75^25^. These Omicron variants are generally milder than earlier strains and may be less likely to cause long‐term complications. Second, COVID‐19 may increase the risk of miscarriage among pregnant women. A systematic review and meta‐analysis indicated that miscarriage was reported in 9.9% of women during their first trimester and 1.2% during their second trimester^26^. Therefore, miscarriage could introduce competing risks and reduce the case numbers in the exposure group, which could distort the results toward the null.

Our analyses showed that COVID‐19 vaccination was associated with a lower risk of GDM. Previously, some studies indicated that COVID‐19 vaccinations might be related to an increased risk of GDM^27^, ^28^, while a systematic review and meta‐analysis summarizing eight studies found no evidence of such associations (pooled OR = 1.15, 95% CI 1.00–1.33)^29^. However, if GDM is considered a manifestation of long COVID, vaccination could indirectly reduce its risk, as receiving vaccination either before or after SARS‐CoV‐2 infection may lower the risk of developing long COVID^30^. However, previous research on COVID‐19 vaccine hesitancy indicates that pregnant women who accept vaccines are more likely to have higher levels of education and to be employed. These socioeconomic factors may be associated with better access to healthcare resources and greater health awareness, contributing to improved maternal care outcomes^31^. Because of this potential healthy volunteer bias, the interpretation of the association between vaccination and GDM should remain cautious^32^.

On the other hand, the risk of incident GDM was also lower among non‐Taiwanese nationalities, which could be explained by a healthy immigrant mother effect in Taiwan, indicating that selective migration and younger maternal age may contribute to favorable outcomes. In a previous population‐based study in Taiwan, it was reported that babies born to immigrant mothers had lower risks of low birth weight, preterm birth, and neonatal mortality compared with those born to Taiwanese mothers^33^.

There are some strengths regarding our study. Our data is population‐based and nationally representative so that we can minimize selection bias. The large sample size further enhances statistical power. Additionally, we also considered the effect of vaccinations on our study, which is not mentioned in the US study^8^. Another key strength is that our case–control study excluded immortal follow‐up time in the analysis, and the results were further confirmed by a cohort design using landmark analysis. Our design and analysis avoid not only immortal follow‐up time but also avoid the converging issue of the log‐binomial model^34^. Therefore, we suggest that a case–control design might be a more straightforward approach when assessing GDM.

Nevertheless, some inherent limitations need to be taken into consideration. First, as mentioned earlier, COVID‐19 has been reported to increase the risk of miscarriage during the first and second trimesters. This creates competing risks and reduces the number of cases in the COVID‐19 exposure group. However, due to limitations in our data, we are unable to assess the impact of early miscarriages occurring before the 24th week of gestation. Future studies should take this potential competing risk into account. Second, COVID‐19 exposure may be misclassified because only people who received COVID‐19 tests and were reported by the healthcare systems would be identified. Although the testing capacity was relatively sufficient in Taiwan due to the delay in community transmission, people who had asymptomatic infections or mild symptoms would still be misclassified into non‐exposure groups, which was reported in previous studies^35^. These undiagnosed COVID‐19 cases may introduce non‐differential misclassification bias, potentially attenuating the observed associations. Third, we could not adjust for antiviral treatments for COVID‐19, such as Paxlovid, because such medication was not approved for pregnant women until May 2022. Lastly, the NHIRD is a claims database that does not include several important covariates, such as BMI, smoking status, family history, laboratory results, and ethnicity. Consequently, our study may be subject to residual confounding.

## CONCLUSION

Our population‐based case–control study found no evidence supporting an association between prior COVID‐19 infection and incident GDM. However, we observed some evidence suggesting that COVID‐19 vaccinations may be associated with a reduced GDM risk. Although COVID‐19 has become endemic with less virulent variants in most countries, healthcare workers should remain vigilant regarding the potential pregnancy‐related impacts of SARS‐CoV‐2. Additionally, policymakers should encourage vaccination among women of childbearing age. Further research is needed to explore COVID‐19‐related complications during pregnancy.

## DISCLOSURE

The authors declare that there are no conflicts of interest.

Approval of the research protocol: N/A.

Informed Consent: N/A.

Approval date of Registry and the Registration No. of the study/trial: N/A.

Animal Studies: N/A.

## Supporting information


**Figure S1.** Illustrating immortal time bias in studies assessing GDM risks.
**Figure S2.** The study DAGs for identifying covariates to be included in the model.
**Figure S3.** The association between COVID‐19 infections and incident GDM using a cohort design with landmark analysis.
**Table S1.** The RECORD statement – checklist of items, extended from the STROBE statement, that should be reported in observational studies using routinely collected health data.
**Table S2.** The distribution of demographic factors of the study population by COVID‐19 history at gestational week 24.
**Table S3.** The association between COVID‐19 infection during pregnancy and incident gestational diabetes stratified by vaccination status.

## Data Availability

Data sharing not applicable to this article as no datasets were generated or analyzed during the current study.

## References

[1] Ely EW , Brown LM , Fineberg HV . Long Covid defined. N Engl J Med 2024; 391: 1753. 10.1056/NEJMsb2408466 PMC11687645 39083764

[2] Xiong X , Lui DTW , Chung MSH , *et al*. Incidence of diabetes following COVID‐19 vaccination and SARS‐CoV‐2 infection in Hong Kong: A population‐based cohort study. PLoS Med 2023; 20: e1004274. 37486927 10.1371/journal.pmed.1004274 PMC10406181

[3] Taylor K , Eastwood S , Walker V , *et al*. Incidence of diabetes after SARS‐CoV‐2 infection in England and the implications of COVID‐19 vaccination: A retrospective cohort study of 16 million people. Lancet Diabetes Endocrinol 2024; 12: 558–568. 39054034 10.1016/S2213-8587(24)00159-1 PMC7617111

[4] Easter SR , Barbieri RL . Medical disorders during pregnancy. In: Loscalzo J , Fauci A , Kasper D , *et al*. (eds). Harrison's Principles of Internal Medicine, 21e. New York, NY: McGraw‐Hill Education, 2022.

[5] Wang H , Li N , Chivese T , *et al*. IDF diabetes atlas: Estimation of global and regional gestational diabetes mellitus prevalence for 2021 by International Association of Diabetes in pregnancy study Group's criteria. Diabetes Res Clin Pract 2022; 183: 9050. 10.1016/j.diabres.2021.109050 34883186

[6] Bengtson AM , Ramos SZ , Savitz DA , *et al*. Risk factors for progression from gestational diabetes to postpartum type 2 diabetes: A review. Clin Obstet Gynecol 2021; 64: 234. 33306495 10.1097/GRF.0000000000000585 PMC7855576

[7] Sweeting A , Hannah W , Backman H , *et al*. Epidemiology and management of gestational diabetes. Lancet 2024; 404: 175–192. 38909620 10.1016/S0140-6736(24)00825-0

[8] Rincón‐Guevara O , Wallace B , Kompaniyets L , *et al*. Association between severe acute respiratory syndrome coronavirus 2 infection during pregnancy and gestational diabetes: A claims‐based cohort study. Clin Infect Dis 2024; 4: ciae416. 10.1093/cid/ciae416 39162200

[9] Hernán MA , Sterne JAC , Higgins JPT , *et al*. A structural description of biases that generate immortal time. Epidemiology 2025; 36: 107. 39494894 10.1097/EDE.0000000000001808 PMC11598638

[10] Daniel S , Koren G , Lunenfeld E , *et al*. Immortal time bias in drug safety cohort studies: Spontaneous abortion following nonsteroidal antiinflammatory drug exposure. Am J Obstet Gynecol 2015; 212: 307.e1–307.e6. 10.1016/j.ajog.2014.09.028 25265406

[11] Hernán MA , Sauer BC , Hernández‐Díaz S , *et al*. Specifying a target trial prevents immortal time bias and other self‐inflicted injuries in observational analyses. J Clin Epidemiol 2016; 79: 70–75. 27237061 10.1016/j.jclinepi.2016.04.014 PMC5124536

[12] Yadav K , Lewis RJ . Immortal time bias in observational studies. JAMA 2021; 325: 686–687. 33591334 10.1001/jama.2020.9151

[13] National Health Insurance Administration Ministry of Health and . National Health Insurance Administration Ministry of Health and Welfare. Taiwan: National Health Insurance Administration Ministry of Health and Welfare, 2022.

[14] National Health Insurance Administration Ministry of Health and Welfare . National Health Insurance Annual Report 2022–2023. Taiwan: National Health Insurance Administration Ministry of Health and Welfare, 2022.

[15] Lin LY , Warren‐Gash C , Smeeth L , *et al*. Data profile: The National Health Insurance Research Database (NHIRD) of Taiwan. Epidemiol Health 2018; 1: e2018062‐0. 10.4178/epih.e2018062 PMC6367203 30727703

[16] The Diabetes Association of the Republic of China (Taiwan) . Clinical Practice Guidelines for Diabetes Mellitus in Pregnancy 2023. 2023 [Internet] [cited 2024 Feb 22] Available from: http://www.endo‐dm.org.tw/dia/direct/index.asp?BK_KIND=56&current=2023%E5%AD%95%E6%9C%9F%E7%B3%96%E5%B0%BF%E7%97%85%E8%87%A8%E5%BA%8A%E7%85%A7%E8%AD%B7%E6%8C%87%E5%BC%95. 10.1016/j.jfma.2019.02.016 30952480

[17] Taiwan Maternal Fetal Medicine Society . Taiwan GDM‐care Guideline. 2025.

[18] Health Promotion Administration . Health Promotion Administration. 2021 [cited 2025 Aug 12]. Expanded Subsidy for Prenatal Examination Services, Effective on July 1. Available from: https://www.hpa.gov.tw/Pages/Detail.aspx?nodeid=1052&pid=14660.

[19] Peng YS , Lin JR , Cheng BH , *et al*. Incidence and relative risk for developing cancers in women with gestational diabetes mellitus: A nationwide cohort study in Taiwan. BMJ Open 2019; 9: e024583. 10.1136/bmjopen-2018-024583 PMC6398720 30796123

[20] Lin MH , Yang AC , Wen TH . Using regional differences and demographic characteristics to evaluate the principles of estimation of the residence of the population in National Health Insurance Research Databases (NHIRD). Taiwan J Public Health 2011; 30: 347–361.

[21] Benchimol EI , Smeeth L , Guttmann A , *et al*. The REporting of studies conducted using observational routinely‐collected health data (RECORD) statement. PLoS Med 2015; 12: e1001885. 26440803 10.1371/journal.pmed.1001885 PMC4595218

[22] World Medical Association Declaration of Helsinki . Ethical principles for medical research involving human subjects. JAMA 2013; 310: 2191–2194. 24141714 10.1001/jama.2013.281053

[23] Lal A , Erondu NA , Heymann DL , *et al*. Fragmented health systems in COVID‐19: Rectifying the misalignment between global health security and universal health coverage. Lancet 2021; 397: 61–67. 33275906 10.1016/S0140-6736(20)32228-5 PMC7834479

[24] Hsieh VCR , Tsai MH , Chiang HC , *et al*. Lessons learned from Taiwan's response to the COVID‐19 pandemic: Successes, challenges, and implications for future pandemics. Eur J Public Health 2025; 35: 153–162. 39566089 10.1093/eurpub/ckae185 PMC11832153

[25] Chen YH , Cheuh YN , Chen CM , *et al*. Epidemiological characteristics of the three waves of COVID‐19 epidemic in Taiwan during April 2022 to march 2023. J Formos Med Assoc 2023; 122: 1174–1182. 37301691 10.1016/j.jfma.2023.05.027 PMC10213295

[26] van Baar JAC , Kostova EB , Allotey J , *et al*. COVID‐19 in pregnant women: A systematic review and meta‐analysis on the risk and prevalence of pregnancy loss. Hum Reprod Update 2024; 30: 133–152. 38016805 10.1093/humupd/dmad030 PMC10905512

[27] Blakeway H , Prasad S , Kalafat E , *et al*. COVID‐19 vaccination during pregnancy: Coverage and safety. Am J Obstet Gynecol 2022; 226: 236.e1–236.e14. 10.1016/j.ajog.2021.08.007 PMC8352848 34389291

[28] Dick A , Rosenbloom JI , Karavani G , *et al*. Safety of third SARS‐CoV‐2 vaccine (booster dose) during pregnancy. Am J Obstet Gynecol MFM 2022; 4: 100637. 35398583 10.1016/j.ajogmf.2022.100637 PMC8988438

[29] Rahmati M , Yon DK , Lee SW , *et al*. Effects of COVID‐19 vaccination during pregnancy on SARS‐CoV‐2 infection and maternal and neonatal outcomes: A systematic review and meta‐analysis. Rev Med Virol 2023; 33: e2434. 36896895 10.1002/rmv.2434

[30] Byambasuren O , Stehlik P , Clark J , *et al*. Effect of covid‐19 vaccination on long covid: Systematic review. BMJ Med 2023; 2: e000385. 10.1136/bmjmed-2022-000385 PMC9978692 36936268

[31] Bhattacharya O , Siddiquea BN , Shetty A , *et al*. COVID‐19 vaccine hesitancy among pregnant women: A systematic review and meta‐analysis. BMJ Open 2022; 12: e061477. 10.1136/bmjopen-2022-061477 PMC9393853 35981769

[32] Delgado‐Rodríguez M , Llorca J . Bias. J Epidemiol Community Health 2004; 58: 635–641. 15252064 10.1136/jech.2003.008466 PMC1732856

[33] Hsieh WS , Hsieh CJ , Jeng SF , *et al*. Favorable neonatal outcomes among immigrants in Taiwan: Evidence of healthy immigrant mother effect. J Womens Health 2011; 20: 1083–1090. 10.1089/jwh.2011.2809 21668384

[34] McNutt LA , Wu C , Xue X , *et al*. Estimating the relative risk in cohort studies and clinical trials of common outcomes. Am J Epidemiol 2003; 157: 940–943. 12746247 10.1093/aje/kwg074

[35] Lin LY , Mulick A , Mathur R , *et al*. The association between vitamin D status and COVID‐19 in England: A cohort study using UK biobank. PLoS One 2022; 17: e0269064. 35666716 10.1371/journal.pone.0269064 PMC9170112

