# Supplementary material for: The association between COVID‐19 and incident gestational diabetes (GDM): A population‐based case–control study of the National Health Insurance Research Database in Taiwan
Source: J Diabetes Investig. 2025 Dec 28;17(3):527–34. doi: 10.1111/jdi.70228 (PMC12950913; doi:10.1111/jdi.70228)
Supplement: Supplementary file 1 — Figure S1. Illustrating immortal time bias in studies assessing GDM risks. Figure S2. The study DAGs for identifying covariates to be included in the model. Figure S3. The association between COVID‐19 infections and incident GDM using a cohort design with landmark analysis. Table S1. The RECORD statement – checklist of items, extended from the STROBE statement, that should be reported in observational studies using routinely collected health data. Table S2. The distribution of demographic factors of the study population by COVID‐19 history at gestational week 24. Table S3. The association between COVID‐19 infection during pregnancy and incident gestational diabetes stratified by vaccination status. [file JDI-17-527-s001.docx]

## **
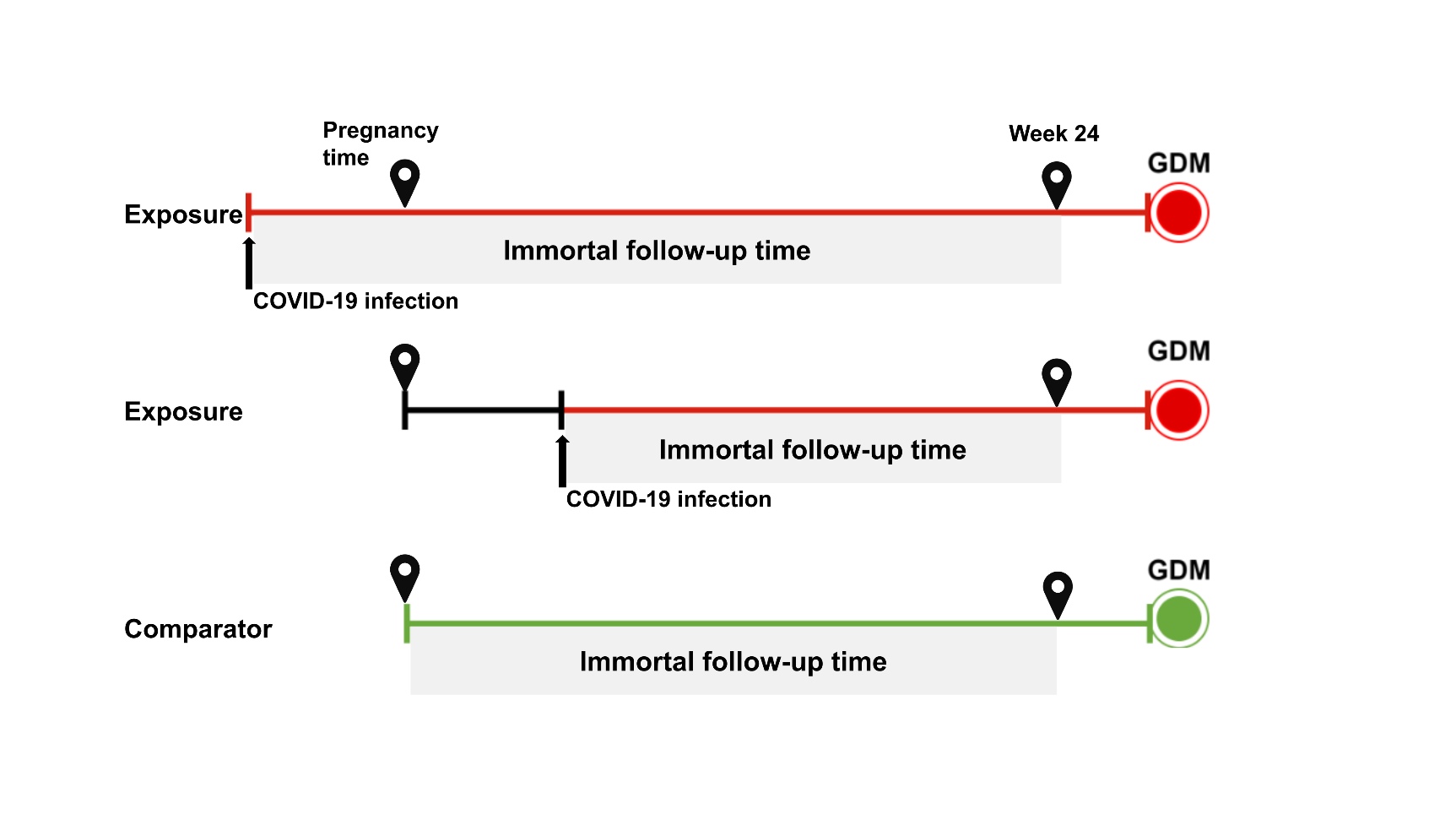
Supplementary materials**

**Supplementary Figure 1.** Illustrating immortal time bias in studies assessing GDM risks

##
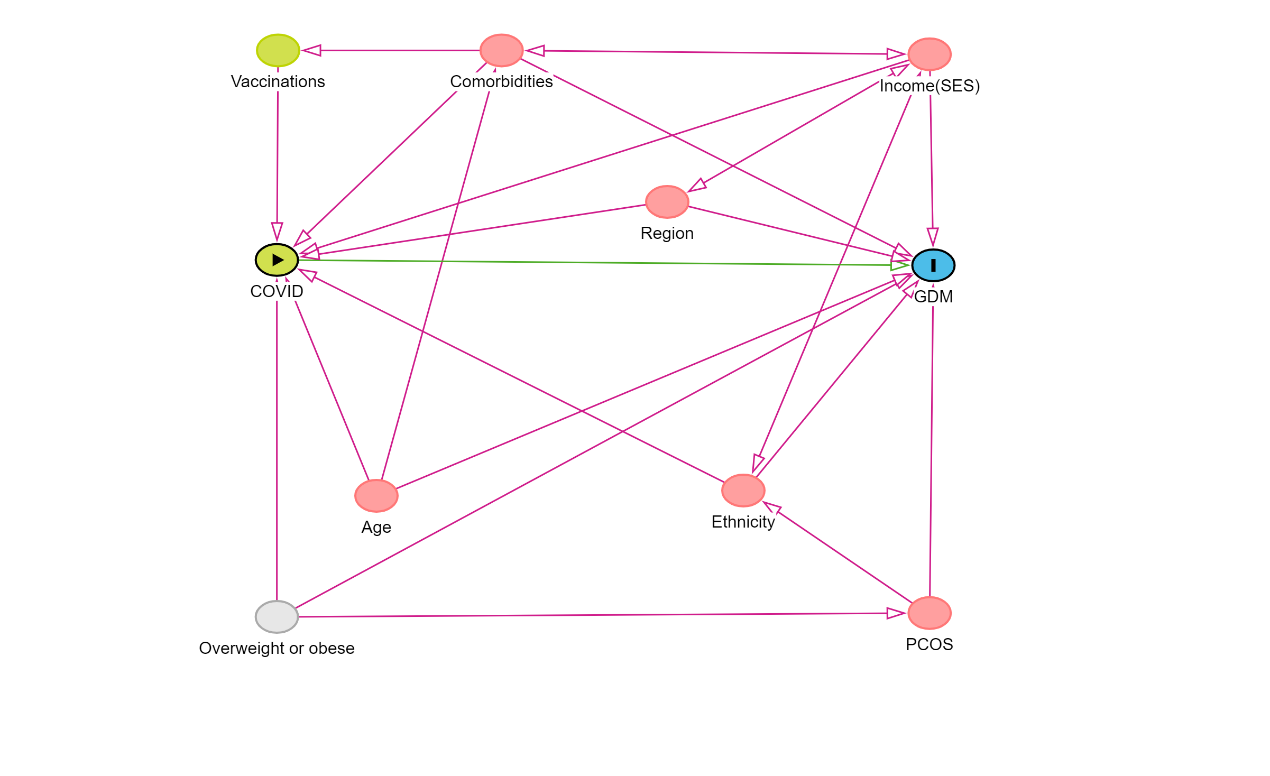
**Supplementary Figure 2.** The study DAGs for identifying covariates to be included in the model.


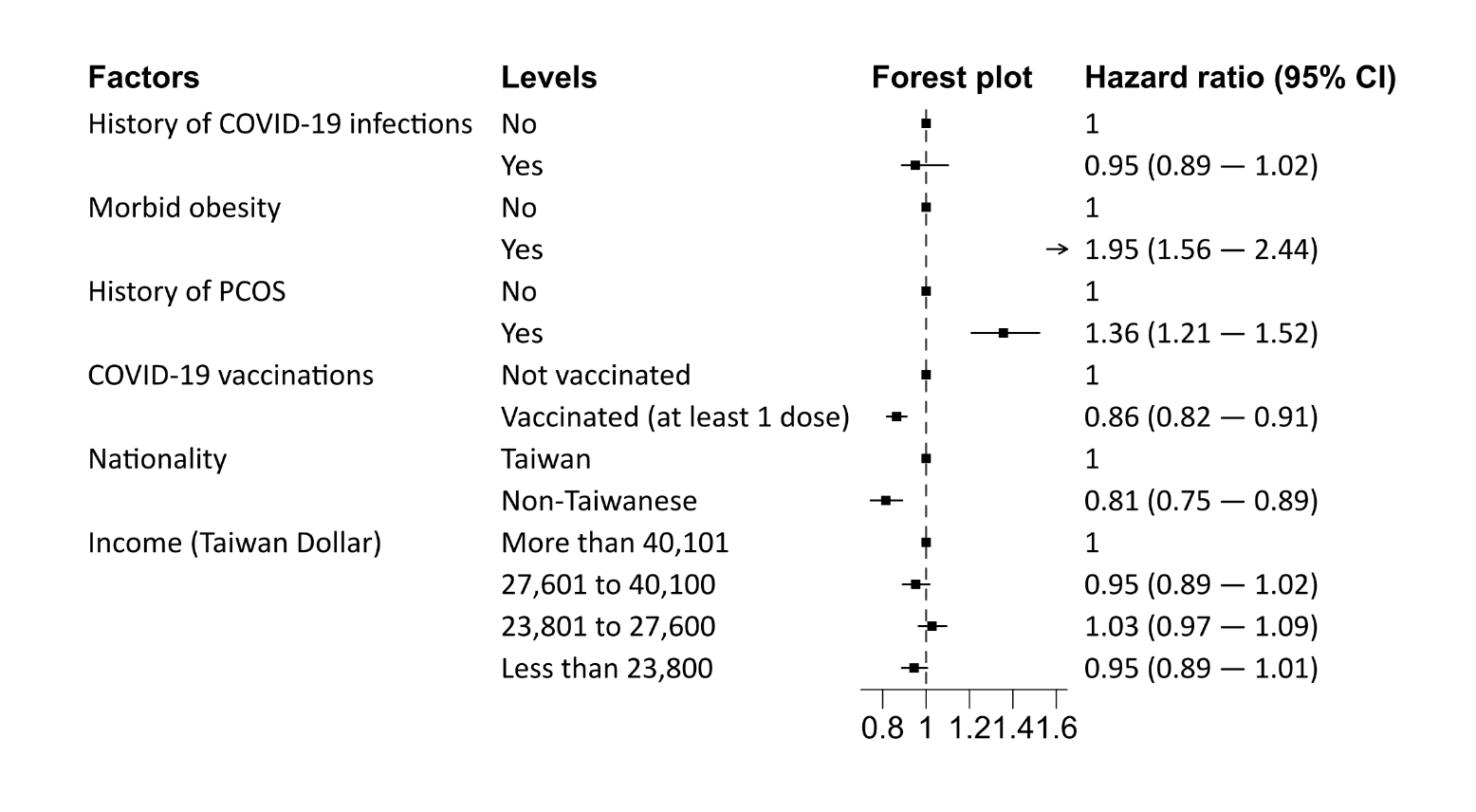
**Supplementary Figure 3.** The association between COVID-19 infections and incident GDM using a cohort design with landmark analysis.

**Supplementary Table 1. The RECORD statement – checklist of items, extended from the STROBE statement, that should be reported in observational studies using routinely collected health data.**

|  | **Item No.** | **STROBE items** | **Location in manuscript where items are reported** | **RECORD items** | **Location in manuscript where items are reported** |
| --- | --- | --- | --- | --- | --- |
| **Title and abstract** | | | | | |
|  | 1 | (a) Indicate the study’s design with a commonly used term in the title or the abstract (b) Provide in the abstract an informative and balanced summary of what was done and what was found | Title page | RECORD 1.1: The type of data used should be specified in the title or abstract. When possible, the name of the databases used should be included.  RECORD 1.2: If applicable, the geographic region and timeframe within which the study took place should be reported in the title or abstract.  RECORD 1.3: If linkage between databases was conducted for the study, this should be clearly stated in the title or abstract. | Title page |
| **Introduction** | | | | | |
| Background rationale | 2 | Explain the scientific background and rationale for the investigation being reported | Page 1—3 |  |  |
| Objectives | 3 | State specific objectives, including any prespecified hypotheses | Page 3 |  |  |
| **Methods** | | | | | |
| Study Design | 4 | Present key elements of study design early in the paper | Page 3 |  |  |
| Setting | 5 | Describe the setting, locations, and relevant dates, including periods of recruitment, exposure, follow-up, and data collection | Page3—4 |  |  |
| Participants | 6 | *(a) Cohort study* - Give the eligibility criteria, and the sources and methods of selection of participants. Describe methods of follow-up  *Case-control study* - Give the eligibility criteria, and the sources and methods of case ascertainment and control selection. Give the rationale for the choice of cases and controls  *Cross-sectional study* - Give the eligibility criteria, and the sources and methods of selection of participants  *(b) Cohort study* - For matched studies, give matching criteria and number of exposed and unexposed  *Case-control study* - For matched studies, give matching criteria and the number of controls per case |  | RECORD 6.1: The methods of study population selection (such as codes or algorithms used to identify subjects) should be listed in detail. If this is not possible, an explanation should be provided.  RECORD 6.2: Any validation studies of the codes or algorithms used to select the population should be referenced. If validation was conducted for this study and not published elsewhere, detailed methods and results should be provided.  RECORD 6.3: If the study involved linkage of databases, consider use of a flow diagram or other graphical display to demonstrate the data linkage process, including the number of individuals with linked data at each stage. | Page 3—4 |
| Variables | 7 | Clearly define all outcomes, exposures, predictors, potential confounders, and effect modifiers. Give diagnostic criteria, if applicable. | Page 4—6 | RECORD 7.1: A complete list of codes and algorithms used to classify exposures, outcomes, confounders, and effect modifiers should be provided. If these cannot be reported, an explanation should be provided. | The final codes will be uploaded to GitHub |
| Data sources/ measurement | 8 | For each variable of interest, give sources of data and details of methods of assessment (measurement).  Describe comparability of assessment methods if there is more than one group | Page 3—4 |  |  |
| Bias | 9 | Describe any efforts to address potential sources of bias | Page 5—6 |  |  |
| Study size | 10 | Explain how the study size was arrived at |  |  |  |
| Quantitative variables | 11 | Explain how quantitative variables were handled in the analyses. If applicable, describe which groupings were chosen, and why | Page 9 |  |  |
| Statistical methods | 12 | (a) Describe all statistical methods, including those used to control for confounding  (b) Describe any methods used to examine subgroups and interactions  (c) Explain how missing data were addressed  (d) *Cohort study* - If applicable, explain how loss to follow-up was addressed  *Case-control study* - If applicable, explain how matching of cases and controls was addressed  *Cross-sectional study* - If applicable, describe analytical methods taking account of sampling strategy  (e) Describe any sensitivity analyses | Page 9 |  |  |
| Data access and cleaning methods |  | .. |  | RECORD 12.1: Authors should describe the extent to which the investigators had access to the database population used to create the study population.  RECORD 12.2: Authors should provide information on the data cleaning methods used in the study. | Page 4 — 6 |
| Linkage |  | .. |  | RECORD 12.3: State whether the study included person-level, institutional-level, or other data linkage across two or more databases. The methods of linkage and methods of linkage quality evaluation should be provided. | Page 4 — 5 |
| **Results** | | | | | |
| Participants | 13 | (a) Report the numbers of individuals at each stage of the study (*e.g.*, numbers potentially eligible, examined for eligibility, confirmed eligible, included in the study, completing follow-up, and analysed)  (b) Give reasons for non-participation at each stage.  (c) Consider use of a flow diagram |  | RECORD 13.1: Describe in detail the selection of the persons included in the study (*i.e.,* study population selection) including filtering based on data quality, data availability and linkage. The selection of included persons can be described in the text and/or by means of the study flow diagram. | Page 7 |
| Descriptive data | 14 | (a) Give characteristics of study participants (*e.g.*, demographic, clinical, social) and information on exposures and potential confounders  (b) Indicate the number of participants with missing data for each variable of interest  (c) *Cohort study* - summarise follow-up time (*e.g.*, average and total amount) | Page 7—8, Table 1 |  |  |
| Outcome data | 15 | *Cohort study* - Report numbers of outcome events or summary measures over time  *Case-control study* - Report numbers in each exposure category, or summary measures of exposure  *Cross-sectional study* - Report numbers of outcome events or summary measures | Page 7—8, Table 1 |  |  |
| Main results | 16 | (a) Give unadjusted estimates and, if applicable, confounder-adjusted estimates and their precision (e.g., 95% confidence interval). Make clear which confounders were adjusted for and why they were included  (b) Report category boundaries when continuous variables were categorized  (c) If relevant, consider translating estimates of relative risk into absolute risk for a meaningful time period | Figure 3 |  |  |
| Other analyses | 17 | Report other analyses done—e.g., analyses of subgroups and interactions, and sensitivity analyses | Supplementary Table 2, Supplementary Figure 3 |  |  |
| **Discussion** | | | | | |
| Key results | 18 | Summarise key results with reference to study objectives | Page 9 |  |  |
| Limitations | 19 | Discuss limitations of the study, taking into account sources of potential bias or imprecision. Discuss both direction and magnitude of any potential bias | Page 12 | RECORD 19.1: Discuss the implications of using data that were not created or collected to answer the specific research question(s). Include discussion of misclassification bias, unmeasured confounding, missing data, and changing eligibility over time, as they pertain to the study being reported. | Page 12 |
| Interpretation | 20 | Give a cautious overall interpretation of results considering objectives, limitations, multiplicity of analyses, results from similar studies, and other relevant evidence | Page 10—11 |  |  |
| Generalisability | 21 | Discuss the generalisability (external validity) of the study results |  |  |  |
| **Other Information** | | | | | |
| Funding | 22 | Give the source of funding and the role of the funders for the present study and, if applicable, for the original study on which the present article is based | Title page |  |  |
| Accessibility of protocol, raw data, and programming code |  | .. |  | RECORD 22.1: Authors should provide information on how to access any supplemental information such as the study protocol, raw data, or programming code. | The study protocol and codes will be available on GitHub |

*Reference: Benchimol EI, Smeeth L, Guttmann A, Harron K, Moher D, Petersen I, Sørensen HT, von Elm E, Langan SM, the RECORD Working Committee. The REporting of studies Conducted using Observational Routinely-collected health Data (RECORD) Statement. *PLoS Medicine* 2015; in press.

*Checklist is protected under Creative Commons Attribution ([CC BY](http://creativecommons.org/licenses/by/4.0/)) license.

**Supplementary Table 2. The distribution of demographic factors of the study population by COVID-19 history at gestational week 24**

|  | **Total** (N = 64,245) | **Had COVID** (N= 12,849) | **Without COVID** (N = 51,396) |
| --- | --- | --- | --- |
| **Mean age at GA24** | 31.7 (5.4) | 31.6 (5.4) | 31.7 (5.4) |
| **Had GDM** |  |  |  |
| No | 56,051 (87.25%) | 11,369 (88.48%) | 44,682 (86.94%) |
| Yes | 8,194 (12.75%) | 1,480 (11.52%) | 6,714 (13.06%) |
| **COVID-19 vaccinations** |  |  |  |
| Not vaccinated | 37,756 (58.77%) | 748 (5.82%) | 37,008 (72.01%) |
| Vaccinated (≥ 1 dose) | 26,489 (41.23%) | 12,101 (94.18%) | 14,388 (27.99%) |
| **Morbid obesity** |  |  |  |
| Not obese | 63,943 (99.53%) | 12,757 (99.28%) | 51,186 (99.59%) |
| Obese | 302 (0.47%) | 92 (0.72%) | 210 (0.41%) |
| **Polycystic ovarian syndrome (PCOS)** |  |  |  |
| No | 62,511 (97.3%) | 12,390 (96.43%) | 50,121 (97.52%) |
| Yes | 1,734 (2.7%) | 459 (3.57%) | 1,275 (2.48%) |
| **Region of residence** |  |  |  |
| Taipei City | 5,685 (8.85%) | 1,137 (8.85%) | 4,548 (8.85%) |
| New Taipei City | 13,900 (21.64%) | 2,780 (21.64%) | 11,120 (21.64%) |
| Keelung City | 1,080 (1.68%) | 216 (1.68%) | 864 (1.68%) |
| Taoyuan City | 8,375 (13.04%) | 1,675 (13.04%) | 6,700 (13.04%) |
| Hsinchu City | 1,255 (1.95%) | 251 (1.95%) | 1,004 (1.95%) |
| Hsinchu County | 1,840 (2.86%) | 368 (2.86%) | 1,472 (2.86%) |
| Miaoli County | 1,170 (1.82%) | 234 (1.82%) | 936 (1.82%) |
| Nantou County | 1,295 (2.02%) | 259 (2.02%) | 1,036 (2.02%) |
| Taichung City | 7,920 (12.33%) | 1,584 (12.33%) | 6,336 (12.33%) |
| Yunlin County | 1,310 (2.04%) | 262 (2.04%) | 1,048 (2.04%) |
| Chiayi City | 555 (0.86%) | 111 (0.86%) | 444 (0.86%) |
| Chiayi County | 980 (1.53%) | 196 (1.53%) | 784 (1.53%) |
| Changhua County | 3,475 (5.41%) | 695 (5.41%) | 2,780 (5.41%) |
| Tainan City | 3,780 (5.88%) | 756 (5.88%) | 3,024 (5.88%) |
| Kaohsiung City | 6,270 (9.76%) | 1,254 (9.76%) | 5,016 (9.76%) |
| Pingtung County | 1,890 (2.94%) | 378 (2.94%) | 1,512 (2.94%) |
| Yilan County | 1,230 (1.91%) | 246 (1.91%) | 984 (1.91%) |
| Hualien County | 1,240 (1.93%) | 248 (1.93%) | 992 (1.93%) |
| Taitung County | 585 (0.91%) | 117 (0.91%) | 468 (0.91%) |
| Penghu County | 205 (0.32%) | 41 (0.32%) | 164 (0.32%) |
| Kinmen County & Lienchiang County (Matsu) | 205 (0.32%) | 41 (0.32%) | 164 (0.32%) |
| **Income (Taiwan dollars)** |  |  |  |
| <23,800 | 19,802 (30.82%) | 3,830 (29.81%) | 15,972 (31.08%) |
| 23,801-27,600 | 16,028 (24.95%) | 3,100 (24.13%) | 12,928 (25.15%) |
| 27,601-40,100 | 14,326 (22.3%) | 3,155 (24.55%) | 11,171 (21.74%) |
| >=40,101 | 14,089 (21.93%) | 2,764 (21.51%) | 11,325 (22.03%) |
| **Original nationality** |  |  |  |
| Taiwan | 59,128 (92.04%) | 12,430 (96.74%) | 46,698 (90.86%) |
| Non-Taiwanese | 5,117 (7.96%) | 419 (3.26%) | 4,698 (9.14%) |

| **Supplementary Table 3: The association between COVID-19 infection during pregnancy and incident gestational diabetes stratified by vaccination status** | | | | |
| --- | --- | --- | --- | --- |
|  | **Vaccinated (≥ 1 Dose)** | | **Not vaccinated** | |
|  | **Partially adjusted model ^1^** | **Fully adjusted model ^2^** | **Partially adjusted model ^1^** | **Fully adjusted model ^2^** |
| COVID-19 infection history |  |  |  |  |
| No | Reference | Reference | Reference | Reference |
| Yes | 0.952 (0.891 to 1.018) | 0.95 (0.889 to 1.015) | 0.964 (0.761 to 1.222) | 0.98 (0.773 to 1.242) |
| Age | 1.009 (1.004 to 1.015) | 1.009 (1.003 to 1.014) | 0.997 (0.993 to 1) | 0.995 (0.991 to 0.998) |
| Morbid obesity |  |  |  |  |
| No | Reference | Reference | Reference | Reference |
| Yes | - | 2.079 (1.594 to 2.712) | - | 1.552 (1.194 to 2.017) |
| History of PCOS |  |  |  |  |
| No | Reference | Reference | Reference | Reference |
| Yes | - | 1.598 (1.432 to 1.784) | - | 1.464 (1.331 to 1.61) |
| Nationality |  |  |  |  |
| Taiwan | Reference | Reference | Reference | Reference |
| Non-Taiwanese | - | 0.985 (0.847 to 1.145) | - | 0.793 (0.749 to 0.84) |
| Socioeconomic status (TWD) |  |  |  |  |
| <23800 | - | 1.007 (0.94 to 1.078) | - | 0.921 (0.879 to 0.965) |
| 23801-27600 | - | 1.014 (0.945 to 1.087) | - | 0.973 (0.928 to 1.02) |
| 27601-40100 | - | 0.951 (0.89 to 1.016) | - | 0.956 (0.91 to 1.004) |
| >=40101 | Reference | Reference | Reference | Reference |

# **Supplementary material: NHIRD COVID and GDM analysis protocol**

### **Analytic codes:**

<https://github.com/cjchen413/Immortal-time-bias-in-COVID-and-GDM/>

### **Research topic:**

Assessing COVID-19 infections and incident GDM among pregnant women in Taiwan

### **Study design:**

1. Main analysis: matched case-control study
2. Sensitivity analysis: cohort study with landmark analysis

### **Database:** NHIRD

| **檔案名稱** | **用途簡介** |
| --- | --- |
| Health07_全民健保承保檔 | 基本人口學資料、Covariates: 投保薪資、投保狀態 |
| Health09_出生通報檔 | 定義懷孕週數、產婦國籍、年齡等 |
| Health10_死因統計檔 | 24週後死亡之產婦 |
| Health102_疾病管制署COVID-19疫苗接種與COVID-19確診、中重症及死亡資料檔 | 定義COVID感染、疫苗接種、是否為重症 |
| Health01_全民健保處方及治療明細檔_門急診 | 看是否有GDM 、PCOS、產檢紀錄 |
| Health02_全民健保處方及治療明細檔_西醫住院 | 看是否有GDM 、PCOS |

### **Timeframe:** 2018-2022

### Research questions:

- Do COVID-19 infections increase the risk of incident gestational diabetes among pregnant adult women?
- What is the impact of immortal time bias in assessing this association?

###

### Study power calculation:

| Power for Unmatched Case-Control Studies | | | | |
| --- | --- | --- | --- | --- |
|  | | | | |
|  | **Input Data** | | | |
| **Two-sided confidence interval (%)** | 95 | | | |
| **Number of cases^1^** | 220276 | | | |
| **Percent of exposure among cases (%)^2,3^** | 10.64 | | | |
| **Number of controls^1^** | 1833030 | | | |
| **Percent of exposure among controls (%)^2,3^** | 89.36 | | | |
| **Odds Ratio** | 0.014 | | | |
|  | | | | |
| **Power based on:** |  |  |  |  |
| Normal approximation | 100% | | | |
| Normal approximation with continuity correction | 100% | | | |
|  | | | | |

Results from OpenEpi, Version 3, open source calculator--PowerCC

1. COVID infection proportion: Rincón-Guevara O, Wallace B, Kompaniyets L, Barrett CE, Bull-Otterson L. Association Between Severe Acute Respiratory Syndrome Coronavirus 2 Infection During Pregnancy and Gestational Diabetes: A Claims-based Cohort Study. Clinical Infectious Diseases [Internet]. 2024 Aug 19 [cited 2024 Oct 10];ciae416. Available from: https://doi.org/10.1093/cid/ciae416
2. GDM source: Su FL, Lu MC, Yu SC, Yang CP, Yang CC, Tseng ST, et al. Increasing trend in the prevalence of gestational diabetes mellitus in Taiwan. Journal of Diabetes Investigation [Internet]. 2021 [cited 2025 Nov 15];12(11):2080–8. Available from: https://onlinelibrary.wiley.com/doi/abs/10.1111/jdi.13595
3. COVID infection trend in Taiwan: Chen YH, Cheuh YN, Chen CM, Kuo HW. Epidemiological characteristics of the three waves of COVID-19 epidemic in Taiwan during April 2022 to March 2023. Journal of the Formosan Medical Association [Internet]. 2023 Nov 1 [cited 2025 Nov 15];122(11):1174–82. Available from: https://www.sciencedirect.com/science/article/pii/S0929664623001961

# Case-control study

### Study framework diagram:

###
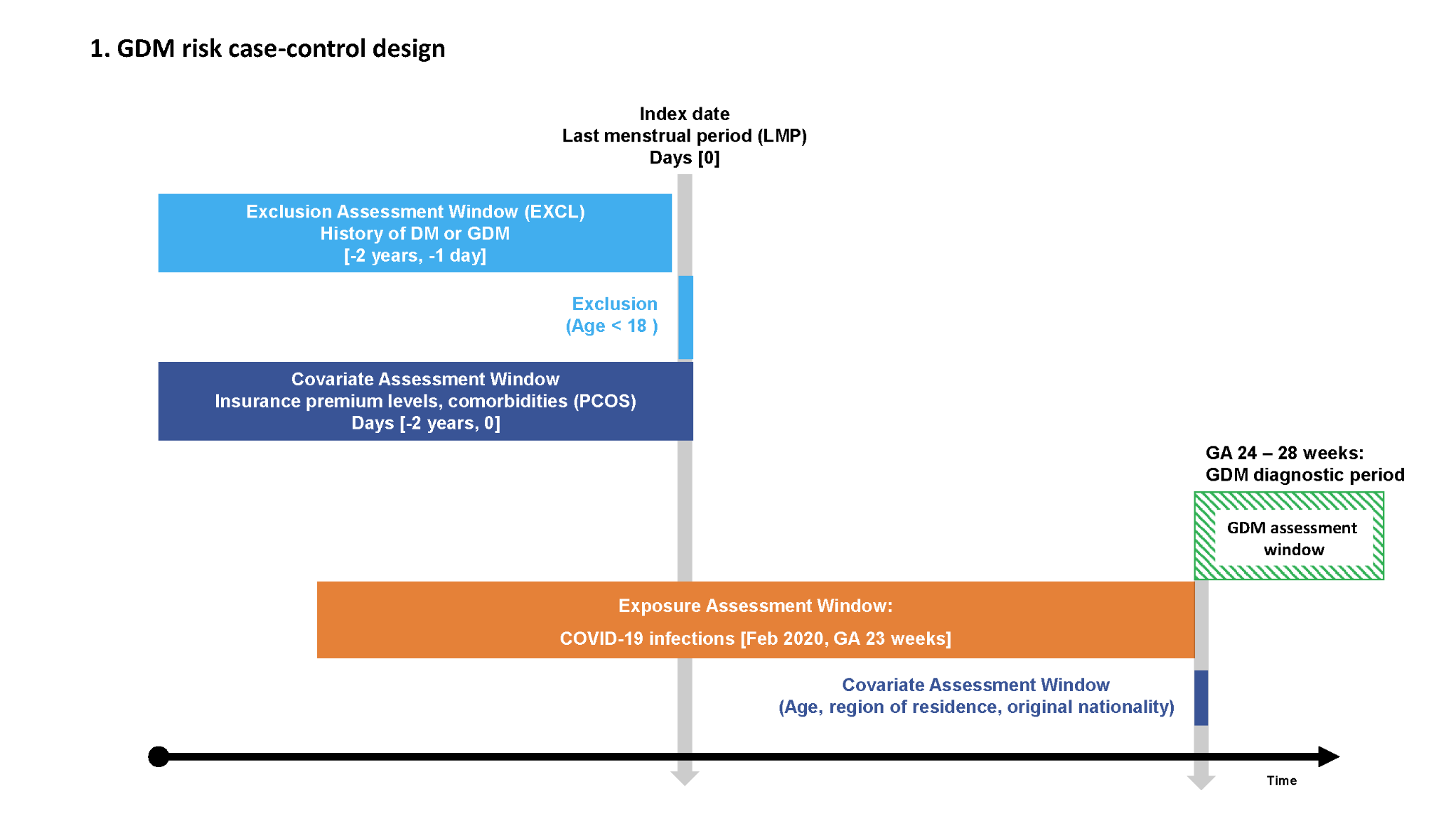


### Study Population Inclusion Criteria:

- Case-control study: adult women, aged >= 18 years old, who had been pregnant for more than 24 weeks.

### Exclusion Criteria:

- Women with a prior diagnosis of type 1 or type 2 diabetes mellitus (DM), diabetic ketoacidosis (DKA), or gestational diabetes mellitus (GDM) in past pregnancies before the index date were excluded.
- Example:

| **Diagnoses** | **ICD-10-CM** |
| --- | --- |
| Personal history of gestational diabetes | Z8632 |
| GDM | O24- |

* See attached codelists

### Defining index date:

- Case-control study: The last menstrual period date (LMP); used Health09 (BIRTH_YM - WEEK)

### Exposure:

- COVID-19 infections, defined as ever had COVID-19 infection before GA 23 weeks.
- Stratify by vaccination status:
  - Not vaccinated
  - Vaccinated: ≥ 1 does
  - Time: received any vaccination before GA 21
- Identified from Health-102 dataset. May use Covid_Confirmation(binary) and Covid_Diagnosed Date (date);
- Hospitalisation due to COVID (a proxy for severity): identify previous hospitalisation due to COVID-19 from *Health_02* data
- Also used ICD-code diagnoses:

| **Diagnoses** |  | **ICD-10-CM** |
| --- | --- | --- |
| COVID-19 新冠肺炎確診 | | U07.1 |
| Other coronavirus as the cause of diseases classified elsewhere  歸類於他處其他冠狀病毒所致的疾病 | | B97.29 |
| Pneumonia due to COVID-19 新冠肺炎導致肺炎 | | J12.82 |
| 新冠肺炎病史 | | Z86.16 |
| Personal history of COVID-19 | |  |
| 後新冠症候群(long COVID) | | U09.9 |
| 成人多系統炎症徵候群Multisystem inflammatory syndrome associated with covid-19 | | U10 - |

### Control/non-cases:

- No GDM (without having GDM diagnoses)
- Covariate matching 1:4: age, gestational weeks, region of residence

### Case/Outcomes:

- Gestational diabetes, defined using the codelist attached

### Covariates:

- Demographic factors: sex, age (on the index date),
  1. Using Health09: region of residence, (original) Nationality
- Clinical factors: obesity (?), polycystic ovarian syndrome (E28.2); history of severe COVID-19 infections (Health102: Covid_ModerateorSevere_Status), vaccination before GA21
- Socioeconomic factors: health insurance premium

### Statistical analysis:

- Descriptive analyses
- Main analysis: logistic regression adjusting for covariates,
  1. Adjustment in the basic model: age, gestational weeks, region of residence
  2. Adjustment in the full model: COVID severity, income, Nationality, vaccination, history of PCOS

### Sensitivity analysis:

| **Sensitivity analysis** | **Justification** |
| --- | --- |
| Re-run the analysis using a cohort design using landmark analysis | By using a cohort design to ensure the temporality, and a landmark analysis further reduced the immortal follow-up time |
| Stratified the analysis according to vaccination status and COVID-19 infection. | To assess the effect modification impact of vaccinations |

#

# Sensitivity analysis: Cohort study using landmark analysis

### Study framework diagram:


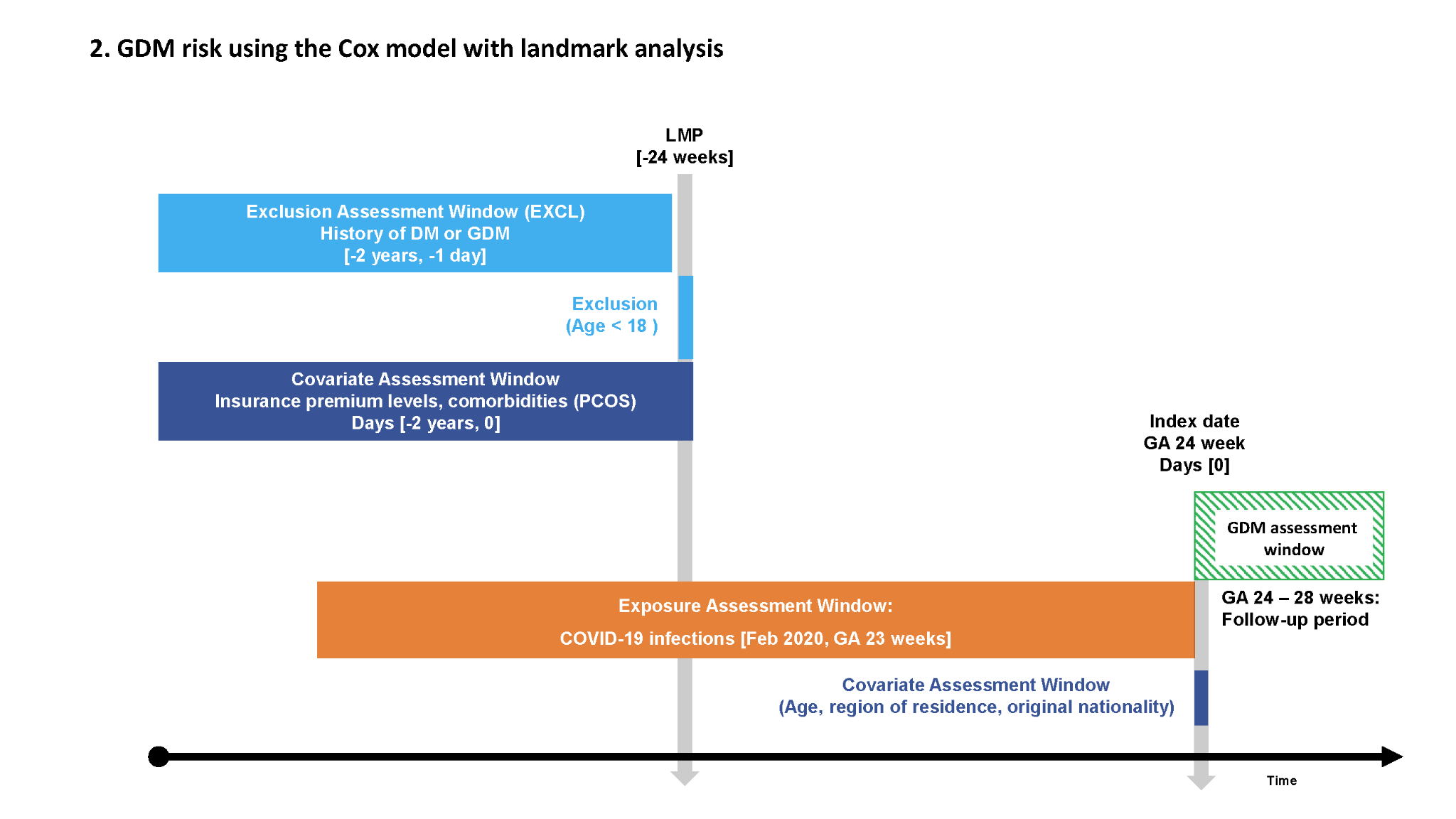


### **Defining index date**:

- Cohort study with landmark analysis: gestational week 24, estimated from Health09
  1. The last menstrual period date (LMP);Health09 (BIRTH_YM - WEEK)
  2. LMP + 24 weeks

### **End of follow-up:**

Which comes first:

1. Had GDM
2. Termination of pregnancy (miscarriage)
3. Death
4. 2022-12-31

### Study population Inclusion Criteria:

- Cohort study with landmark analysis: adult women, aged >= 18 years old, who had been pregnant for more than 24 weeks.

### Exclusion criteria:

- The same as the case-control study.

### Exposure:

- Had a history of COVID-19 infection before GA 23
- The same as the case-control study

### **Comparator:**

- No COVID-19 infection history before GA 23, matching for age, region

### **Outcome**:

- Incidence of GDM, the same as the case-control study

### Statistical analysis:

- Minimal adjusted model: Poisson regression model adjusting for age, region
- Full adjusted model: Poisson regression model adjusting COVID severity, income, Nationality, vaccination, and history of PCOS

#

# Planned outputs:

**Tables:**

**Table 1: (Case-control) The distribution of demographic factors of the study population**

|  | Total, N = | Had GDM, N= | Without GDM, N= |
| --- | --- | --- | --- |
| Mean age at LMP |  |  |  |
| Mean gestational week at GDM diagnosis |  |  |  |
| Region of residence |  |  |  |
| (請簡單列出) |  |  |  |
| Nationality |  |  |  |
| Missing |  |  |  |
| Taiwan |  |  |  |
| Non-Taiwanese |  |  |  |
| …. |  |  |  |
| Obesity |  |  |  |
| Not obese |  |  |  |
| Obese |  |  |  |
| Polycystic ovarian syndrome |  |  |  |
| No |  |  |  |
| Yes |  |  |  |
| COVID-19 infections: |  |  |  |
| Ever had COVID-19 (total) |  |  |  |
| History of COVID-19 before LMP |  |  |  |
| History of COVID-19 after LMP |  |  |  |
| Hospitalised due to (severe) COVID |  |  |  |
| No |  |  |  |
| Yes |  |  |  |
| COVID-19 vaccinations |  |  |  |
| Not fully vaccinated |  |  |  |
| Fully vaccinated |  |  |  |
| Income-based insurance premium |  |  |  |

#### Table S1: (Cohort using landmark analysis) The distribution of demographic factors of the study population (by COVID-19 history at GA 24)

|  | Total, N = | Had COVID, N= | Without COVID, N= |
| --- | --- | --- | --- |
| Mean age at GA24 |  |  |  |
| Region of residence |  |  |  |
| (請簡單列出) |  |  |  |
| Nationality |  |  |  |
| Missing |  |  |  |
| Taiwan |  |  |  |
| Non-Taiwanese |  |  |  |
| …. |  |  |  |
| Obesity |  |  |  |
| Not obese |  |  |  |
| Obese |  |  |  |
| Polycystic ovarian syndrome |  |  |  |
| No |  |  |  |
| Yes |  |  |  |
| COVID-19 infections: |  |  |  |
| Hospitalised due to (severe) COVID |  |  | - |
| No |  |  | - |
| Yes |  |  | - |
| COVID-19 vaccinations |  |  |  |
| Not fully vaccinated |  |  |  |
| Fully vaccinated |  |  |  |
| Income-based insurance premium |  |  |  |
| Had GDM |  |  |  |
| No |  |  |  |
| Yes |  |  |  |
